# Supplementary material for: Prosocial correlates of transformative experiences at secular multi-day mass gatherings
Source: Nat Commun. 2022 May 27;13:2600. doi: 10.1038/s41467-022-29600-1 (PMC9142525; doi:10.1038/s41467-022-29600-1)
Supplement: Supplementary file 1 — Supplementary Information [file 41467_2022_29600_MOESM1_ESM.pdf]

Supplementary Information for:

Prosocial Correlates of Transformative Experience at Secular Multi-Day Mass Gatherings

Daniel A. Yudkin, Annayah Prosser, S. Megan Heller, Kateri McRae, Alek Chakroff, & Molly J. Crockett

Table of Contents

1. Supporting Analyses......3
1.1 Sampling......3
1.1.1 Test-Retest Reliability......3
1.1.2 Data Collection......4
1.2. Drug use.......5
1.3. Moral Expansion......8
1.3.1. Correlation between monetary and temporal moral expansion.......8
1.3.2. Correlation between self-report moral expansion measure and an incentivized moral expansion measure.......10
1.4. Longitudinal Analyses......14
1.5. Expecting and desiring transformative experiences......17
1.5.1. Visualization.......19
1.5.2. Immediate Follow-up Imputation.......20
1.6. Supplemental study: transformative experience at a "virtual" mass gathering......21
1.7. Epistemic Transformative Experience......27
1.8. Qualities of Transformative Experience......27
1.9. Previous Attendance......29
1.10. Other Aspects of Transformative Experience......30
1.10.1. Extent......31
1.10.2. Valence.......31
1.10.3. Moral transformation.......31
1.11. Other Model Specifications......31
1.12. Group identity fusion.......32
1.13. Predictors of Transformative Experience......33
1.13.1. New friends.......34
1.13.2. Giving and receiving gifts.......34
1.13.3. Dancing (behavioral synchrony).......35
3.1. Event Descriptions......36
3.2. Demographic Information......37

4. Full Survey Materials......41

1. Supporting Analyses

1.1 Sampling

1.1.1 Test-Retest Reliability

Our multi-wave sampling strategy enabled us to examine the test-retest reliability of our measures of transformative experience, universal connectedness, and moral expansion over time. Specifically, we used longitudinal analysis to examine the degree to which the measures collected in the pretest phase, and in the onsite phase, correlated with those same measures collected in the immediate follow-up. The results showed high test-retest reliability for all measures (all *p*s < .001; see Supplementary Table 1).

Supplementary Table 1. *Test-retest correlation between pre-test or onsite measures and the same measures taken 0-4 weeks after attendance.*

	Pre-test & immediate follow-up			Onsite & immediate follow-up		
	r	*n*	*p*	*r*	*n*	*p*
Universal connectedness	.55	173	< .001	.64	102	< .001
Moral Expansion	.54	133	< .001	.69	88	< .001
Generosity	.33	183	< .001	.15	62	.23
Transformative Experience				.40	107	< .001

1.1.2 Data Collection

Our data collection strategy sought to collect portions of our total overall desired sample across the duration of each event in order to ensure a wide distribution of days insight and therefore facilitate analyses with time onsite as a predictor variable of subjective transformation and prosocial orientation. Supplementary Figure 1 shows the distribution of participants collected on each day.

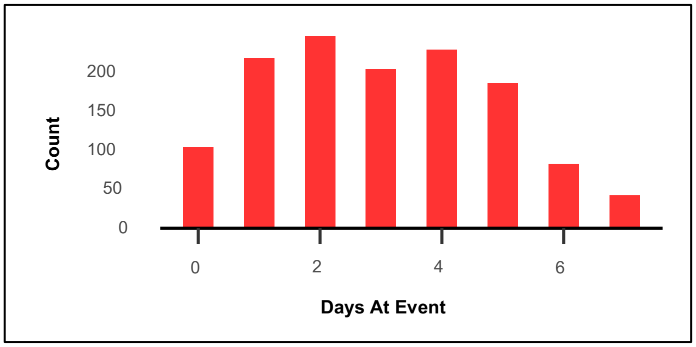

Supplementary Figure 1. Distribution of self-reported days at event when experiment was administered.

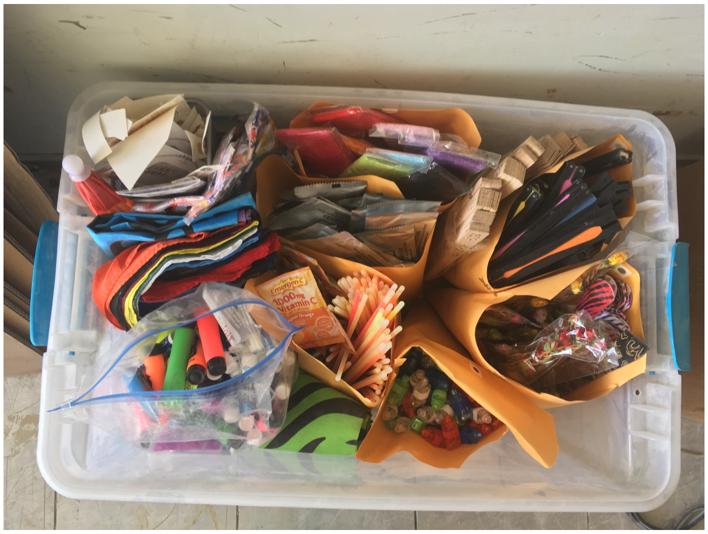

Supplementary Figure 2. Picture of “mystery prize” box featuring various desirable objects to eventgoers.

1.2. Drug use.

Common perception of many of the multi-day mass gatherings we surveyed is that they are a site of prevalent psychoactive substance use. Therefore, it was important to determine the degree to which the use of such substances was related to, or even responsible for, the outcomes we observed. Elsewhere we have reported the mood-enhancing effects of psychedelic substance use and its relationship to transformative experience and universal connectedness[1]. We find that psychedelic use is associated with positive affect, an effect sequentially mediated by transformative experience and universal connectedness. Here we test how psychedelic substance use related to anticipating a transformative experience, as well as the prosocial outcomes tested (i.e., moral expansion and generosity).

1.2.1. Prevalence. Supplementary Table 2 shows the overall use of psychoactive substances onsite. To determine the number of people who did not use any psychoactive substances, we computed two values: first, the percent of participants who said they used no substances whatsoever, and second the percent of participants who said they used no substances controlled by the US government (i.e., no substances except alcohol).

Supplementary Table 2. *Frequency of substance use in onsite sample.*

Substance	% reported using onsite
Alcohol	81
Cannabis	52
Psychedelics	28
Euphorics	25
Stimulants	22
Narcotics	3
No controlled	39
None	13

1.2.2. Psychedelics and anticipation. First, we tested the relationship between drug use and anticipation of transformative experience (i.e., expectations and desires). This was based off the possibility that drug use might have changed people's expectations or caused their experiences to go out of their control. Based off prior research showing that psychedelics (but not other substances) predicted transformative experiences at mass gatherings, and in order to avoid inflated alpha levels stemming from multiple significance tests, we focused our tests on the use of psychedelics (hallucinogens). As specified in the main paper, we entered a model that specified self-reported use of psychedelic substances onsite as either -.5 or .5, then tested whether it predicted desires and expectations for transformative experience, controlling for all incidental variables. Results showed that people who used psychedelics reported both greater desires, $B = 0.36$, $SE = 0.14$, $t(1192) = 2.61$, $p = 0.009$, and greater expectations, $B = 0.35$, $SE = 0.13$, $t(1179) = 2.73$, $p = 0.006$, of transformation. This shows that people who used psychedelics were more likely to both expect and desire to have a transformative experience.

Next we tested whether psychedelic substances interacted with expectations and desires to predict self-reported transformative experience. As previously reported, there was a main effect of psychedelic substance use on transformative experience, $B = 0.44$, $SE = 0.12$, $t(1183) = 3.60$, $p < 0.001$. However no interactions with expectations or desires emerged, both *p*s > .5.

1.2.3. Psychedelics and qualities of transformation. We wanted to see whether psychedelic substance use was more strongly associated with certain types of transformative experience than others. To do this, we conducted a series of linear regressions controlling for incidental variables with each transformation quality as the dependent measure and extracted the beta weight of psychedelic substance use, which reflected the additional degree of each transformation that could be expected for someone who had used psychedelics onsite.

Confidence intervals were Bonferroni-corrected to account for multiple tests. Results (seen in Supplementary Figure 3 below) showed that, with the exception of feeling connected to something larger including "community, culture, history," psychedelic-substance users showed an increased likelihood of reporting each quality of transformative experience (all ps < .01). In particular, psychedelic-substance users were more likely to say they perceived something new about reality or about themselves, or feel more spiritually connected to "a higher power, nature, God."

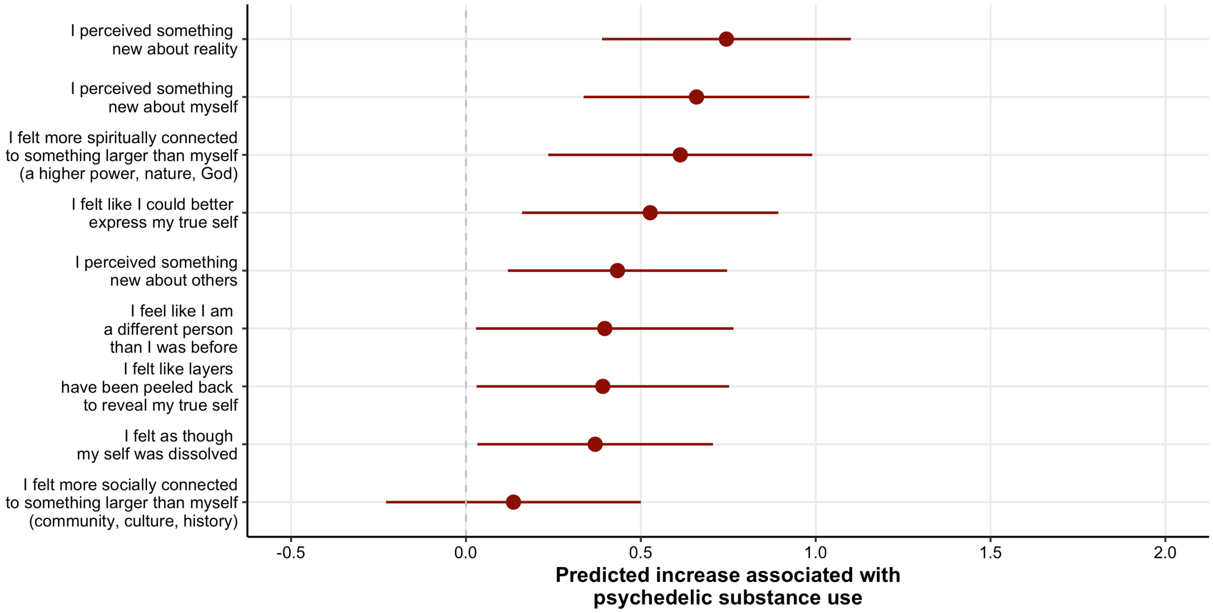

Supplementary Figure 3. The predicted increase of each type of transformative experience among onsite users of psychedelic substances (approximately 28% of a total sample of 1,215 independent observations). Points reflect the beta coefficient obtained from a linear model with each transformation type as the dependent variable and the binary-coded psychedelic substance use variable as the predictor, controlling for all incidental variables. Error bars indicate Bonferroni-corrected 95% confidence intervals.

1.2.4. Psychedelics and prosocial outcomes. Next, we examined the drug use × anticipation interaction on universal connectedness, moral expansion, generosity, respectively. As previously reported, there was a main effect of psychedelic substance use on universal

connectedness, $B = 0.36$, $SE = 0.11$, $t(1128) = 3.37$, $p = 0.001$. However, there were no main effects or interactions between psychedelic substance use and moral expansion or generosity, all *p*s > .2. Finally, we, we tested a model examining the interaction of transformative experience and psychedelic substance use on prosocial outcomes. No interactions emerged, all *p*s > 2. Overall, this analysis suggests that, while psychedelic substance use is strongly predictive of self-reported transformative experiences and experiences of social connection, it does not appear to predict shifts in moral expansion or generosity.

1.3. Moral Expansion

1.3.1. Correlation between monetary and temporal moral expansion. To establish the validity of our "time-based" measure of social discounting (i.e., spending time doing favors for socially close vs. distant social targets) relative to the previously established "money-based" measure, we conducted a study to determine the correlation between these measures. We recruited a sample of 50 participants through Amazon's Mechanical Turk (17 male, 33 female, $M_{age} = 29.5$, $SD = 7.57$). Participants were asked to complete both the time-based and money-based versions of the social discounting measure. They were first provided the instructions, "Some people are more socially close to you than others," and then asked to provide the names of representatives for each of the following social distances: 1, 2, 3, 4, 10 and 20. They then answered the time-based set of questions and the money-based set of questions in random order as applied to these targets.

For the money-based measure, participants were instructed to imagine that someone had given them $100, and they had the opportunity to give any amount of money (from $0 to $100) to each of the persons they had listed. They were instructed to treat each question as a separate case. For the time-based measure, they were asked to imagine they had 14 hours of free time, and

to indicate how much of that time they would be willing to spend doing a personal favor for each of the persons. The persons at distances 50 and 100 were simply called “Person 50” and “Person 100,” respectively.

We then computed the correlation between responses to the money-based versus the time-based measure. As noted in the primary manuscript, and in accordance with past research[2], we obtained for each person an estimate of their overall social discounting by calculating the area under the curve (AUC). AUC was calculated by plotting the respective social distances of each of the targets against the amount of time or money each participant indicated they would give to that target, and then calculating the total area under the curve. AUC was log-transformed to account for non-normal distribution. Below we plot side-by side the distributions of the money-based and time-based measures. Then we show the correlation between the two measures. As shown in Supplementary Figure 4, a robust correlation ($r = .71$, $p < .001$) emerged for the time-based versus money-based measures. This finding suggests that people’s social discount rate, as applied to money-based measures, are closely associated with the novel measure that we developed of time-based social discounting, and supports the methodological validity of our approach.

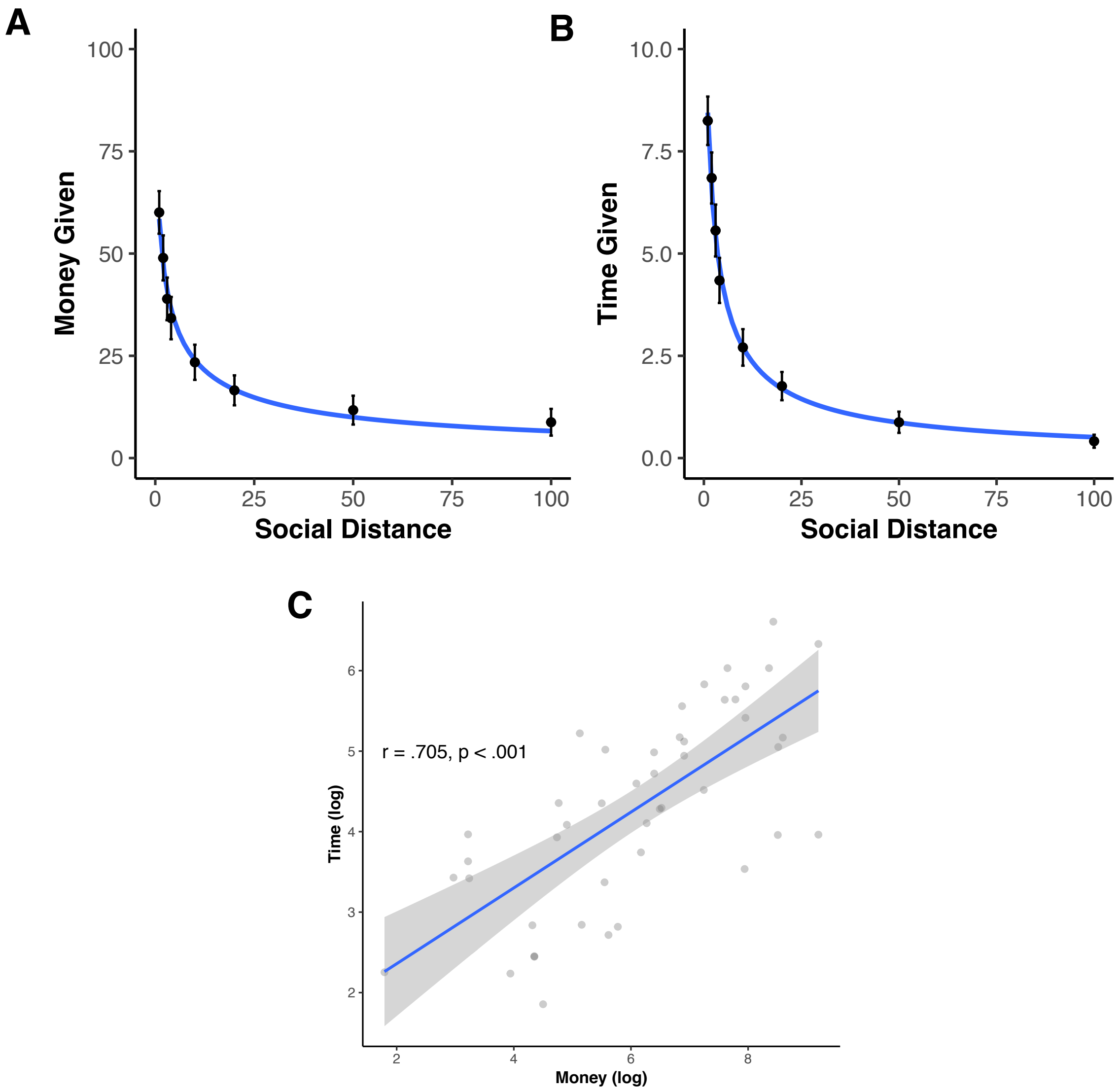

Supplementary Figure 4. Panels A and B: rates in the time-based social discounting measures are similar to those involving money-based rates (n = 50). Money-based and time-based discount rates follow established hyperbolic curves given by the function y ~ $V/(1 + k*x^s)$, where V represents the undiscounted value of the reward (i.e., 14), y the discounted value and k a parameter representing degree of social discounting (i.e. the steepness of the curve). Panel C: scatterplot of area under the curve of money-based versus time-based measures of social discounting. Error bars indicate *SEM*; ribbon 95% CI.

1.3.2. Correlation between self-report moral expansion measure and an incentivized moral expansion measure. One limitation of our main study is that our measure of moral expansion was not incentivized. While this wouldn't necessarily explain the effects outlined in the main paper, it does raise the question of their external validity. We conducted an additional

follow-up experiment to address this issue. We developed an incentivized measure of moral expansion that captured behavioral preferences in charitable donations to near versus distant targets. Specifically, participants had an opportunity to allocate portions of a real, limited resource (a gift of $100) to either themselves or the Red Cross organization of a near or distant country. By creating a task that paralleled the structure of the original moral expansion task, while at the same time including a financial incentive, we hoped to determine whether participants' level of moral expansion on the self-report measure could predict their giving behavior in their incentivized measure.

1.3.2.1. Participants. We recruited 319 participants (146 male, 162 female, $M_{\text{age}} = 40.7$, $SD = 15.1$) from Prolific, an online data collection platform, to take part in a study on social preferences.

1.3.2.2. Method. Participants were told that they would be taking part in a research study looking at how people make social judgments and decisions. After agreeing to participate, participants were asked to indicate their "feelings towards different countries." Specifically, they were told:

> *Some countries may feel more "socially close" to you than others, regardless of how geographically close or far away those countries are. For example, if you were American, you might feel closer to the United Kingdom than to Belize, even though Belize is geographically closer to the U.S., because these countries share a cultural history and language. To help you visualize how socially close various countries are, we could construct the diagram below.*
>
> *Now we would like you to think of specific examples of countries with different social distances. In the spaces below please write the name of a country at approximately each of the following social distances from you. To help you with this task, we have provided a map below.*

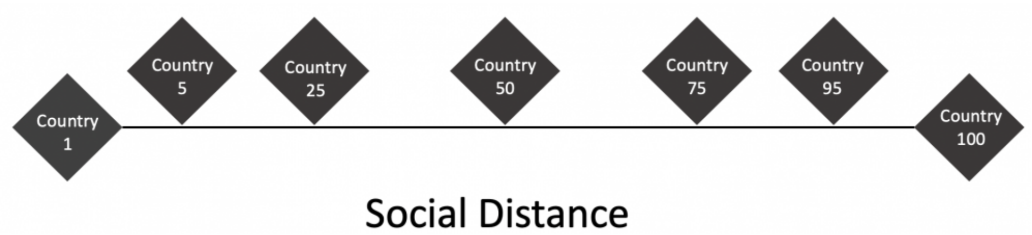

Participants indicated countries for the following distances: 1, 2, 3, 5, 10, 20, 50, and 100 (the same distances that were included in the temporal moral expansion task). After indicating these countries, participants were provided the following instructions:

> *In the following activity you are going to have the opportunity to distribute money to several different Red Cross organizations.*
>
> *Red Cross is a humanitarian organization that provides emergency assistance, disaster relief, and disaster preparedness education to countries around the world.*
>
> *The way this works is as follows:*
>
> - *On the following pages, you will see a list of different Red Cross organizations.*
> - *For each Red Cross organization you see, you will be asked to allocate $100 between yourself and that organization.*
> - ***This choice matters:*** *we will chose one participant's decision at random and pay the money out according to that response.*
> - *The person who is chosen will be contacted and paid whatever amount they do not allocate to the Red Cross organization.*
> - *For example, if your decision was selected in which you allocated $40 to Red Cross, then you would receive $60.*
> - *Please treat each choice as a separate case.*

In randomized order, participants also completed our non-incentivized moral expansion measure of giving time that was used in the studies reported in the main manuscript. After completing both these measures, participants were thanked and debriefed. In an online preregistration[3] we predicted that "there will be a positive relationship between temporal moral expansion (measured onsite) and an incentivized measure of the same construct."

1.3.2.3. Analysis and Results. First, we calculated the area under the curve (AUC) for both the temporal and the incentivized version of the moral expansion measure. Then we

inspected the correlation between these measures. Results (seen in Supplementary Figure 5) showed a significant correlation, $r(284) = .30$, $p < .001$. Results showed that the incentivized moral expansion measure followed roughly the same pattern as the temporal one, albeit with wider error bars, possibly due to the statistical noise introduced through the sense of need that people may attribute to different countries.

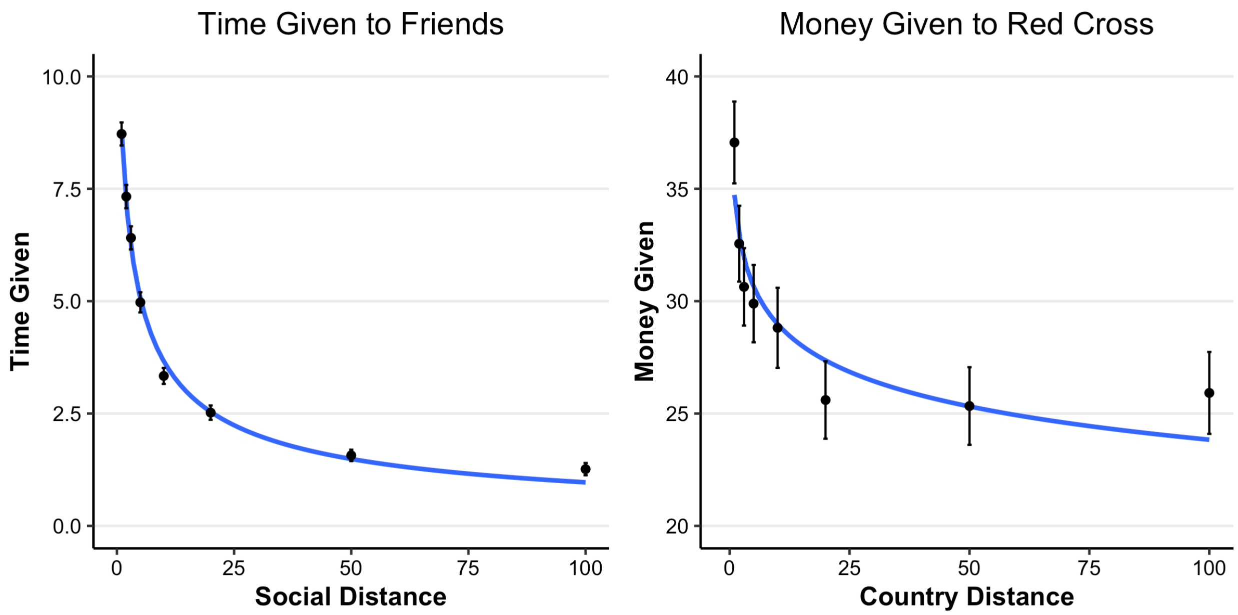

Supplementary Figure 5. Comparison of the moral expansion curve obtained from the temporal (original) versus incentivized measure ($n = 319$). Results of an analysis of the area under the curve (AUC) for both measures yielded a significant correlation, $r(284) = .30$, $p < .001$. Error bars indicate *SEM*.

1.3.2.4. Discussion. The results of this study provide evidence that self-reported moral expansion is significantly correlated with moral expansion as captured by an incentivized measure. Because it shows that people who self-report a higher willingness to give time to more socially distant (versus close) others are more likely to give money to more distant (versus close) countries, it serves as indirect support for the claim that the responses people provided on our self-reported

temporal measure of moral expansion reflect genuine levels of moral expansion. At the same time, this evidence should not be treated as conclusive. It does not guarantee that someone with high self-reported temporal moral expansion would show high levels of moral expansion in an incentivized measure. Support for this claim would need to come from a measure that tests generosity to near versus distant others directly.

1.4. Longitudinal Analyses

The findings reported in the main text demonstrate correlational relationships between transformative experiences, universal connectedness and moral expansion at mass gatherings. In this section, we report supplemental analyses exploring the directionality of these relationships. First, it is possible that universal connectedness and moral expansion predispose people to having transformative experiences, rather than the other way around (as we have hypothesized). To address this possibility, we used longitudinal analyses to examine whether universal connectedness and moral expansion assessed prior to mass gathering attendance predicted transformative experiences reported in the immediate follow-up ("pre-post" within-subjects dataset). Results showed that neither pre-event moral expansion, $B = 0.03$, $SE = 0.09$, $t(175) = 0.32$, $p = 0.746$, nor pre-event universal connectedness, $B = 0.14$, $SE = 0.13$, $t(162) = 1.05$, $p = 0.297$, positively predicted reports of transformative experiences in the immediate follow-up. Next, we tested whether onsite levels of universal connectedness and moral expansion predicted reports of transformative experiences immediately following attendance, controlling only for within-subject autocorrelations of onsite transformative experience (to account for the possibility that onsite prosociality had been affected by onsite transformative experience). Results showed that neither onsite universal connectedness, $B = 0.15$, $SE = 0.11$, $t(103) = 1.38$, $p = .169$, nor onsite moral expansion, $B = 0.16$, $SE = 0.16$, $t(103) = 0.99$, $p = .326$, predicted transformative

experiences in the immediate follow-up. Thus, we do not find evidence that higher levels of preexisting universal connectedness or moral expansion predispose people to report transformative experiences. However, the conclusions we can draw from these analyses are limited due to the small sample sizes, and must be corroborated in future studies with larger samples. That is, it may be the case that pre-event levels of universal connectedness and moral expansion predispose people towards transformative experiences at mass gatherings, and these analyses were underpowered to detect this relationship.

Next, we tested for evidence in the longitudinal sample that transformative experience onsite was related to prosocial behavior following attendance. Analyses showed that transformative experience reported onsite was associated with generosity immediately following attendance, despite the small sample size, $B = 0.49$, $SE = 0.20$, $t(71) = 2.39$, $p = 0.02$. This is consistent with the idea that transformative experience results in more generous behavior. Transformative experience onsite also predicted universal connectedness immediately following attendance, $B = 0.16$, $SE = 0.08$, $t(88) = 2.03$, $p = 0.045$. Yet universal connectedness did not predict generosity either onsite or immediately following attendance, both ps > .2. Thus there is insufficient evidence to suggest that transformative experience increases generosity by increasing universal connectedness.

Next we examined whether transformative experience and universal connectedness reported onsite predicted moral expansion 0-4 weeks after attendance. To do this, we constructed a mediation model that tested the significance of the indirect effect from onsite transformative experience and universal connectedness to moral expansion in the immediate follow-up. Results revealed a marginally significant indirect effect from onsite transformative experience to follow-up moral expansion via onsite universal connectedness, $B = .057$, $SE = .033$, $p = .084$, CI_{95}[-.008,

.123], $n = 140$. Component paths analysis showed a significant association between transformative experience and universal connectedness onsite, $B = 0.13$, $SE = 0.07$, $t(132) = 1.90$, $p = 0.06$, and from onsite universal connectedness to follow-up moral expansion, $B = 0.21$, $SE = 0.07$, $t(76) = 3.21$, $p = 0.002$. However, the direct effect from transformative experience to moral expansion was not significant, $p = .754$. Thus evidence for a longitudinal effect of transformative experience on subsequent moral expansion is mixed.

Next, using the pre-post within-subjects dataset, we created difference scores that reflected changes in moral expansion and universal connectedness before versus after attending. We then constructed a model that tested the degree to which transformative experience reported immediately following attendance predicted these cross-temporal changes in universal connectedness and moral expansion, controlling for all incidental follow-up variables and for baseline (pre-event) universal connectedness and moral expansion. No significant effects emerged for moral expansion, $B = 0.03$, $SE = 0.06$, $t(116) = 0.52$, p = .603. However, a significant effect did emerge for universal connectedness, $B = 0.19$, $SE = 0.06$, $t(136) = 3.36$, p = .001, showing that the greater participants' reported transformative experience 0-4 weeks after attending mass gatherings, the greater the difference in their level of universal connectedness before versus after the event. This provides longitudinal evidence of a relationship between transformative experience and changes in universal connectedness.

Ultimately, we found evidence of a significant relationship between universal connectedness onsite and subsequent moral expansion, but the indirect path from transformative experience onsite to subsequent moral expansion did not reach significance ($p = .084$). And while neither universal connectedness nor moral expansion measured prior to mass gathering attendance did not significantly predict transformation onsite, it is possible an association would

emerge with greater sample sizes. More research is needed to determine whether transformative experiences cause changes in universal connectedness and moral expansion.

1.5. Expecting and desiring transformative experiences

We investigated the extent to which mindset (expectations and desires for transformative experiences) moderated the relationships between transformative experiences, universal connectedness, and moral expansion. Both expecting (B = 0.16, SE = 0.04, t(1174) = 4.40, p < .001) and desiring (B = 0.23, SE = 0.03, t(1176) = 7.83, p < .001) a transformative experience positively predicted the likelihood of having one. However, anticipation was not a necessary precondition of having a transformative experience. If expectations or desires were necessary, then we should not observe any participants who report low expectations/desires but high levels of transformative experience. However, 13.3% of people who said they did "not at all" expect to have a transformative experience (n = 366) ultimately indicated feeling "absolutely" transformed, and 38.0% of these people felt at least "somewhat" transformed. Similarly, 9.8% of people who did "not at all" desire to have a transformative ultimately said they were "absolutely" transformed, and 33.3% said they were at least "somewhat" transformed. Moreover, of the people who responded "not at all" to *both* expecting and desiring a transformative experience (21.7% of the whole sample), 9.4% "absolutely" had a transformative experience and 27.1% at least "somewhat" did. Insofar as these are people who come in with no expectations or desires whatsoever, these results show that prior mindset is not a necessary requirement for ultimately reporting a transformative experience.

We also find evidence suggesting that neither expectations nor desires are sufficient for having a transformative experience. In our onsite data, 15.6% of people who said that they "absolutely" *expected* to have a transformative experience (n=102; 8.4% of total sample), and

13.6% of people who said that they “absolutely” *desired* to have a transformative experience (n=186; 15.2% of total sample), indicated that they did not even “somewhat” have one (see SOM 1.5.1 and Supplementary Figure 6 for additional information regarding the relationship between expectations and desires and transformative experience).

These findings were further corroborated by results of an additional study at a “virtual” mass gathering following the cancellation of Burning Man 2020 due to the COVID-19 pandemic (see SOM 1.6 and Supplementary Figure 7), showing that people reported greater desire for, but less actual, transformation at the virtual event relative to attending Burning Man in person. Overall, then, while expectations and desires were positively associated with having a transformative experience, our data suggest they were neither necessary nor sufficient for having one. Because we control for these mindset variables in all of our key analyses, we suggest that mindset cannot entirely account for the observed relationships between transformative experiences, universal connectedness, and moral expansion.

Nevertheless, as a further test of whether expectations and desires for transformative experience could account for these relationships, we examined whether these variables independently predicted universal connectedness and moral expansion. If we found that expectations or desires positively predicted these prosocial measures, this would provide evidence for an alternative explanation for our findings: that anticipating transformative experiences, rather than having them, is associated with an expanded moral orientation. Results, however, did not support this alternative explanation: neither expecting nor desiring a transformative experience was positively associated with universal connectedness or moral expansion, all ps > .40 (see SOM Appendix B, Tables 3 and 4).

Next, to test for moderating effects of expectations and desires on the relationship

between time, transformative experience, universal connectedness, and moral expansion, we tested the statistical interaction between expectations/desires and each of these variables, controlling for all incidental variables. Results showed no significant interactions (all *p*s > .10), suggesting that the relationships described in the primary model above were not moderated by mindset.

1.5.1. Visualization. Here we plot the relationship between expected, desired, and experienced transformation among the on-site participants across all events. The purpose is to observe the frequency with which participants fall in the "off-diagonals" of these relationships, in order to determine the degree to which transformative experience depends on anticipation. In the Supplementary Figure below (Supplementary Figure 6), we highlight the participants who answered either below or above "somewhat" in their expectations and desires for transformation, and vice versa in their experienced transformation, and provide descriptive labels for each of these categories (e.g., "Unexpected transformers"). The positive correlations between reported transformative experience and both expectations and desires corroborate the findings, reported in the main paper, that the greater people's anticipation of having a transformative experience, the greater their likelihood of having one. Yet the considerable minority of the sample (i.e., 26% and 17%, respectively) that falls in the off-diagonal areas of the plots shows that desires and expectations are neither necessary nor sufficient for having a transformative experience.

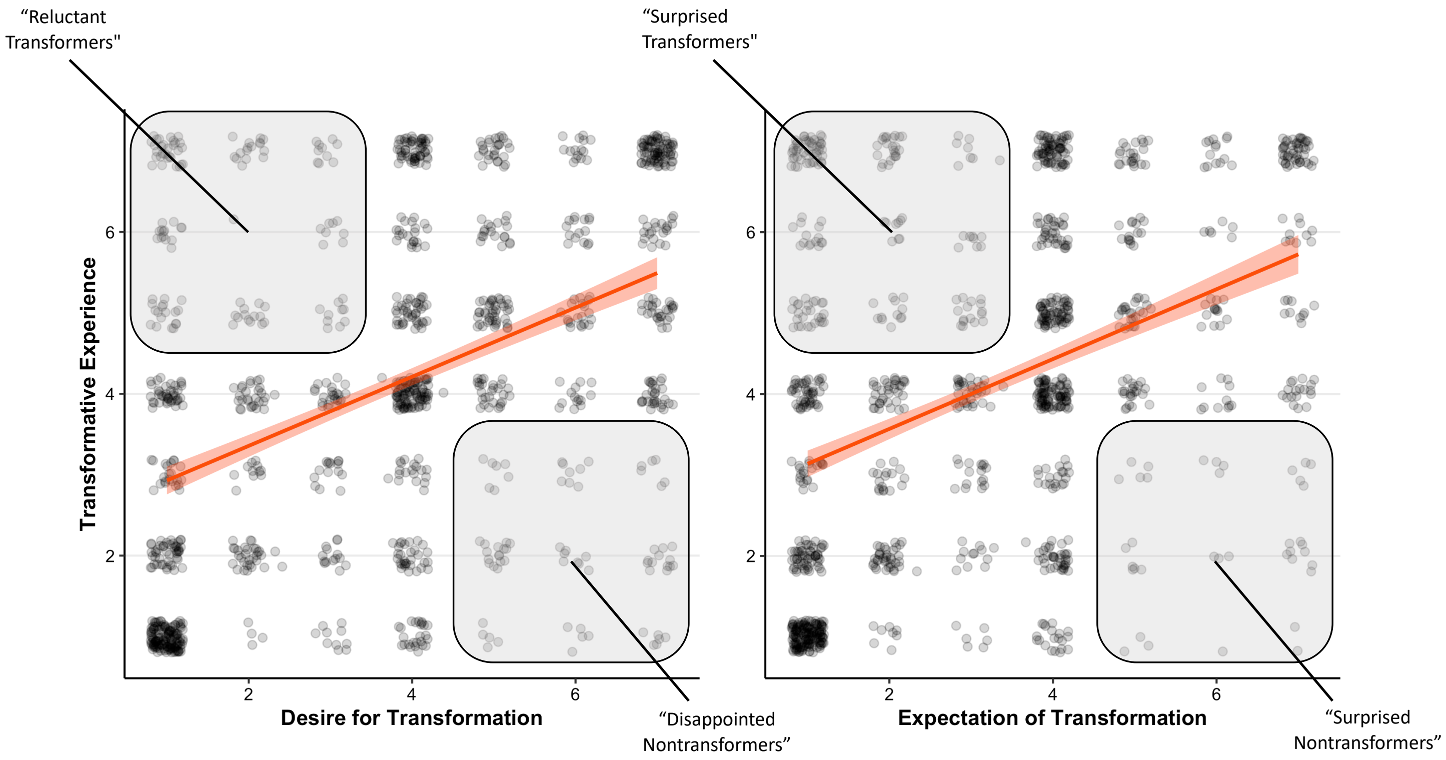

Supplementary Figure 6. Jittered scatterplots of the relationship between desiring and expecting, and the magnitude of actual reported, transformative experience. Proportions of the entire sample are as follows: Reluctant Transformers: 20.0%, Disappointed Nontransformers: 6.2%, Surprised Transformers: 13.8%; Surprised Nontransformers: 3.1%.

1.5.2. Immediate Follow-up Imputation. Measures of transformative anticipation were not collected in the online immediate followup of Burning Man 2016 due to experimenter oversight. Because this was the largest source of immediate follow-up data, (N = 1,759), performing strict listwise deletion on the regression analyses for this measure resulted in an unacceptably small sample sizes for these analyses (N = 107). Thus we dropped "expectation" and "desire" for transformative experience from the reported immediate follow-up analyses. To ensure that these results remained robust when including these in the mediation model testing the relationship between transformative experience, universal connectedness, and moral expansion, we performed two supplementary analyses. First, we re-ran the model testing the significance of the relationship using just the expectation and desire variables as controls (method 1). Then, we

used a data imputation procedure to estimate the missing data, and re-ran the analyses (method 2). The imputation procedure was implemented using the *mice* package in R[4], which is based on fully conditional specification. We used a predictive mean matching method, which reduces bias by drawing real values sampled from the data. The model was set at 5 iterations. The algorithm then runs the prespecified regression model on each dataset separately and pools the results.

Results showed no change in the significance values of the relationship between transformative experience, universal connectedness, and moral expansion from those reported in the paper. Using method 1, we found that transformative experience significantly predicted universal connectedness, $B = 0.12$, $SE = 0.01$, $t(5516) = 9.41$, $p < .001$, which significantly predicted moral expansion $B = 0.18$, $SE = 0.08$, $t(76) = 2.38$, $p = .020$. The indirect effect from transformative experience to moral expansion through universal connectedness was significant, $B = .034$, $SE = .014$, $p = .018$, CI_{95}[.006, .061]. Similarly, using method 2, we found that transformative experience significantly predicted universal connectedness, $B = 0.10$, $SE = 0.01$, $t(150) = 11.17$, $p < .001$, which significantly predicted moral expansion, $B = 0.11$, $SE = 0.02$, $t(4.73) = 4.74$, $p = .010$. The indirect effect was also significant, $B = .014$, $SE = .002$, $p < .001$, CI_{95}[.008, .014]. Thus including anticipation results in no substantive changes to any of the reported effects.

1.6. Supplemental study: transformative experience at a “virtual” mass gathering

In May of 2020, the Burning Man organization announced the cancellation of that year’s physical event, normally held in the Black Rock Desert of central Nevada, due to the COVID-19 pandemic. Instead, it held a “Virtual Burn”: an immersive virtual reality experience that would allow participants the opportunity to explore a virtual online space, complete with art exhibits, virtual talks, and workshops. The difference was that people would participate from their own

homes. A *New York Times* op-ed claimed that the event "maintains much of the energy, abundance and wonder of the real thing."[5] This virtual event presented a unique opportunity to gather additional information on the relationships between expectations, desires, and transformative experiences. Unlike in-person mass gatherings, where we observed high incidence of transformative experiences as well as their anticipation, we reasoned that participants might expect that a virtual event would be unlikely to generate the same kinds of transformative experiences that arise at an in-person mass gathering. Nevertheless, we thought it plausible that many attendees would still strongly desire to feel transformed by participating in the virtual event. If this were the case, we might observe a dissociation between desiring a transformative experience and actually having one in a situation where transformative experiences were unlikely and unexpected, thus further supporting our claim that merely desiring a transformative experience is not sufficient to have one.

Our approach, therefore, was to collect data on transformative experiences at the Virtual Burn and compare it to results from a comparable sample in our original dataset. We selected Burning Man 2016 as the comparison group because it was our most recent onsite sample collected at Burning Man.

1.6.1. Procedure. In collaboration with the Burning Man Census, we prepared a survey that included many of the same questions we had asked participants in our onsite studies. Participants were recruited to the study through the "Virtual Portal" of the Burning Man Census. Participants were told they would be asked to "share your experiences" at the Virtual Burn and in return would have the chance to learn some preliminary results of the research. We supplemented this sample by distributing the survey in Jack Rabbit Speaks, an online newsletter for the Burning Man community. After participants clicked on the link leading to the survey, an

experimenter positioned against a virtual background at the same Burning Man location where the original study had been conducted greeted participants and walked them through the informed consent (as we had done in our field studies). Once they had agreed to participate, participants responded to a series of questions about their experiences at the Virtual Burn so far, the number of events they had participated in, as well as a subset of the questions we presented to participants onsite (i.e., universal connectedness, moral expansion, demographics, mood, group fusion, and transformative experience). After participating, participants engaged again with the experimenter, who explained to them the purpose of the study as well as some preliminary findings of the project.

1.6.2. Results. A total of 98 participants (51 men, 42 women, 5 fluid/other) agreed to participate in this experiment, from which 78 (42 men, 35 women, 1 fluid/other) provided enough data to be analyzed (i.e., reported the extent of their transformative experience). While this number is lower than some of the other samples we collected, it nevertheless allowed us to explore some of the differences in people's experiences at virtual versus onsite events.

In order to determine how participants' experiences at the virtual event differed from their experiences onsite, we sought to compare the responses given to the virtual survey with the most appropriate comparison group obtained from the onsite sample. We identified this as the Burning Man 2016 sample, because this consisted of attendees of the same event who answered the same questions as the virtual attendees (the 2015 Burning Man survey questions deviated somewhat, most notably in the moral expansion measure, which measured money instead of time). The comparison sample thus consisted of 350 participants (200 men, 142 women, 8 other/fluid).

First, we sought confirmation that the two samples were largely comparable

demographically. There were no differences in gender, religiosity, income, or education, all *p*s > .2. There was a difference in age, with the virtual sample (M = 45.2, SD = 13.6) being older than the onsite sample (M = 36.7, SD = 11.9), $t(107) = -5.09$, $p < .001$. However, we note that in our main analyses age did not predict likelihood of having a transformative experience.

Next we examined differences in the degree to which onsite versus virtual attendees expected versus desired to be transformed. In order to account for possible differences in demographics and other variables, in this and all subsequent analyses we control for gender, age, income, education, religiosity, mood, and time at event. (In order to render time at event, which was measured in different increments, comparable across samples, we computed a "ranked time" score that ordinally positioned participants relative to each other within each collection phase.) Results showed that, while onsite attendees had greater *expectations* of being transformed at the event than virtual attendees, B = -0.97, SE = 0.22, $t(370) = -4.42$, $p < 0.001$, virtual attendees reported a greater *desire* to be transformed than onsite attendees, B = 1.15, SE = 0.24, $t(370) = 4.81$, $p < 0.001$, see Supplementary Figure 7.

Next we tested whether virtual versus onsite attendees reported differences in transformative experience. If the Burning Man attendee population is inherently predisposed to report feeling transformed, or if desiring a transformative experience is sufficient for having one, then we would not expect to see differences in the levels of reported transformation between the virtual and onsite events. However, results showed that there were significantly higher levels of transformative experience reported onsite than virtually, B = -0.59, SE = 0.28, $t(369) = -2.09$, $p = 0.038$, (see Supplementary Figure 7). Moreover, we find that at both the virtual event, $t(74) = 4.47$, $p < .001$ and the onsite event, $t(342) = 8.96$, $p < .001$, participants ultimately reported feeling more transformed than they expected. The finding that virtual attendees reported higher

desires for transformative experience but significantly lower rates of actual transformation than onsite attendees raised the question of how desires for transformation related to actual transformation. To further understand this, we examined proportions of participants who ultimately felt transformed but did not initially report a desire for transformation. Onsite, 16% of the people who reported “not at all” desiring a transformative experience ended up feeling “absolutely” transformed. At the virtual event this proportion was 0%.

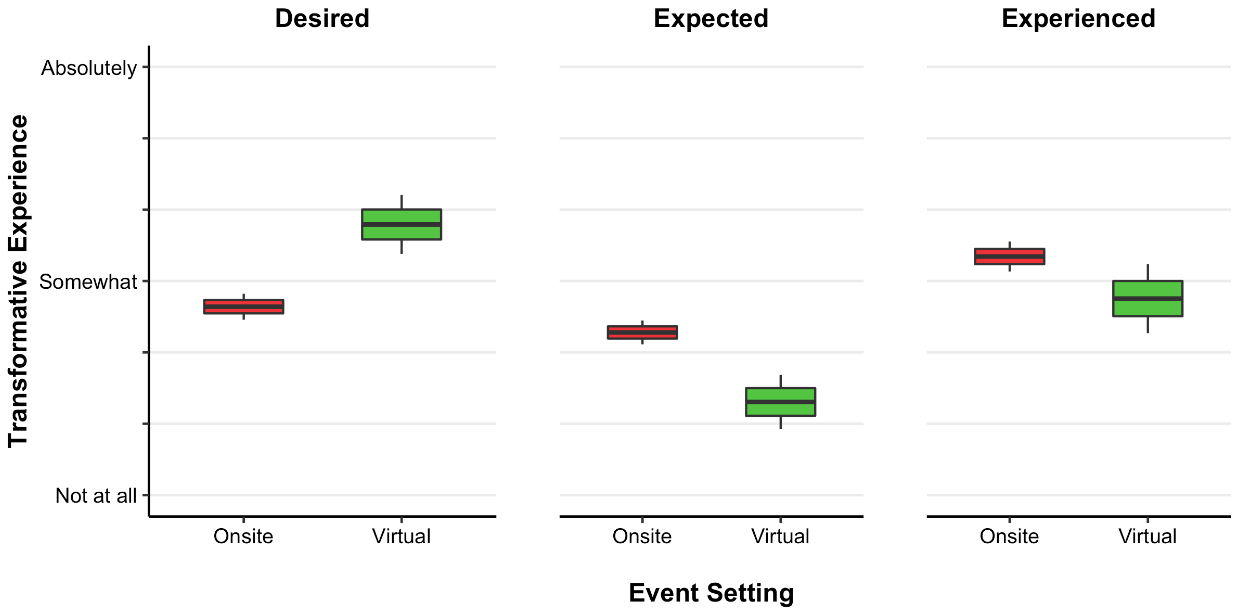

Supplementary Figure 7. Desired, expected, and experienced transformation at the onsite versus virtual Burning Man events predicted from linear regression models controlling for participant demographics (total n = 430). Participants at the virtual event reported greater desire but less actual transformative experience than those at the onsite event. By contrast, participants both expected and experienced greater transformation at the onsite than virtual event. However, experienced transformation exceeded participants’ expectations at both events. Box centers represent mean, edges *SEM*, whiskers 95% CI.

1.6.3. Discussion. The results of this supplementary study provide further evidence about the relationship between anticipated and actual transformative experience. Participants reported greater desire, but less actual transformative experience at the virtual than the in-person event.

By contrast, expectations followed reality more closely, providing additional evidence that expectations play a significant role in predicting people's degree of transformative experience. But at both virtual and on-site events, participants end up reporting that they felt more transformed than they expected. Overall, this data suggest that expectations and desires play an important role in the degree of transformation that people end up experiencing. Indeed, significant correlations between these constructs suggest that people's preexisting mindset at mass gatherings into is an important predictor of transformative experience. However, these data also help support the conclusion, outlined in the main paper, that anticipation is neither necessary nor sufficient for having a transformative experience.

1.6.4. Limitations. The purpose of this study was to assess differences in the level of transformative experiences reported among Burning Man attendees at a virtual versus in-person event. Our results did show such differences, suggesting that characteristics of the sample population are not solely responsible for the high rates of transformative experience reported onsite. At the same time, there are a large number of other factors that varied between the groups that limit the conclusions that can be drawn from this study, including drug use, behavioral synchrony (or any physical motion), sleep deprivation, physical hardship, physical contact with others, dancing, singing, shared food and rituals around eating and food preparation, etc. Thus it is impossible to specify what, exactly may have led to the greater prevalence of transformative experiences at the onsite gathering. Further research will be needed to better understand the psychological differences between virtual and in-person events.

1.7. Epistemic Transformative Experience

In this section, we rerun the primary mediation analyses reported in the main text with epistemic transformative experience. First, we verified that the pattern of increase in transformative experience over time, and lasting sense of transformation, was evidence for epistemic transformation. As seen in Supplementary Figure 8, the pattern was largely consistent, although reports of epistemic transformation were less prevalent than subjective transformative experience.

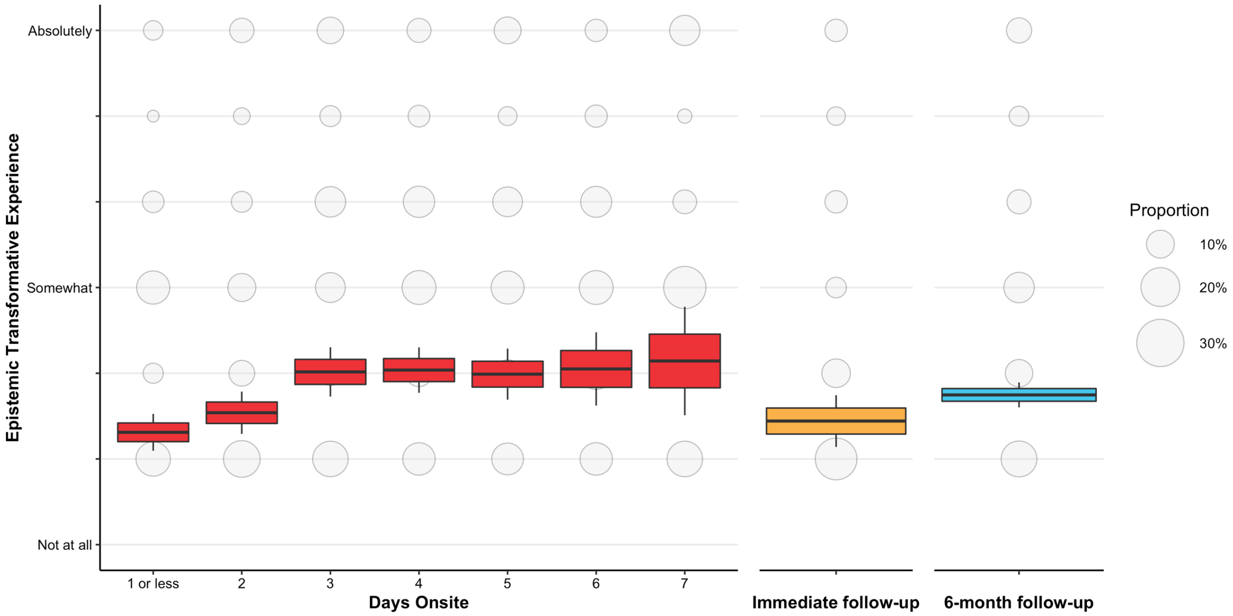

*Supplementary Figure 8***.** Self-reports of epistemic transformative experience increase over time and persist up to six months following attendance (n = 1,215). Boxplot centers indicate average reported transformative experience at each time point; box centers indicate mean; edges *SEM*; whiskers 95% CI. Bubbles reflect the proportion within each timepoint reporting each level of epistemic transformative experience.

1.8. Qualities of Transformative Experience

We assessed a variety of qualities of transformative experiences via thirteen follow-up questions.

Supplementary Table 3. Variable name and wording of each of the qualities of transformation assessed onsite.

**Variable Name**	**Question Wording**
Myself	I perceived something new about myself
Others	I perceived something new about others
Reality	I perceived something new about reality
SociallyConnected	I felt more socially connected to others
SpirituallyConnected	I felt more spiritually connected to something larger than myself (a higher power, nature, God)
Larger	I felt more socially connected to something larger than myself (community, culture, history)
SelfDissolved	I felt as though my "self" was dissolved
PeeledBack	I felt like layers have been peeled back to reveal my true self
TrueSelf	I felt like I could better express my true self
Different	I feel like I am a different person than I was before

In exploratory analyses, we examined the change in each of these qualities over time onsite. Supplementary Figure 9 shows the relationship between each quality of transformation and time, and shows that all qualities of transformation increase significantly over time, with the exception of feeling as though one could express one's true self.

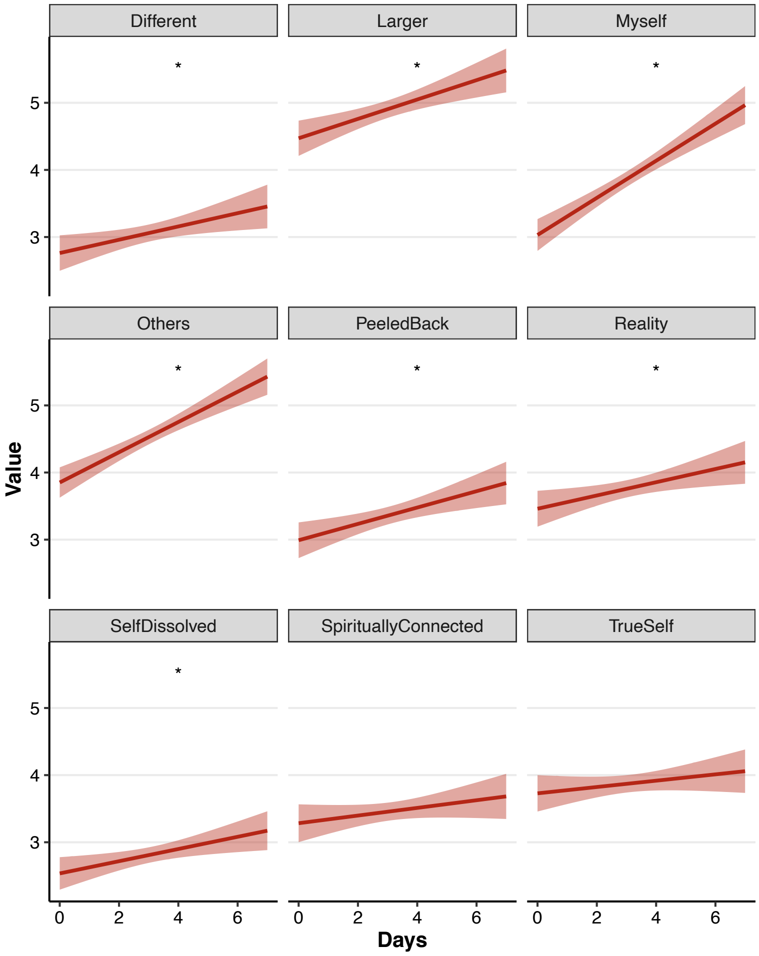

Supplementary Figure 9. Relationship between days onsite and self-reported prevalence of each quality of transformative experience. *$p < .05$.

1.9. Previous Attendance

If attending an event for the first time exposes someone to insights they might not have had before, it is possible that first-time attendees would experience greater transformation than veterans. To test this question, we performed a series of analyses using previous attendance (first-timer versus veteran) as the primary predictor of transformation, controlling for all incidental variables. This variable indicated whether or not participants had attended that event at least once in the past. Results showed a main effect onsite in which, contrary to expectations,

veteran attendees reported greater transformative experience than first-timers, *B* = 0.79, *SE* = 0.10, *t*(1175) = 7.99, p < .001. To better understand this effect, we tested a model that included an attendance by days-at-festival interaction as a predictor. The interaction was significant, *B* = -0.24, *SE* = 0.06, *t*(1173) = -4.33, p < .001. Probing the effect at each level of attendance revealed that while veterans showed a significant increase in transformative experience over time, *B* = 0.09, *SE* = 0.04, *t*(494) = 1.97, p = .049, first-timers felt significantly more transformed with each passing day (*B* = 0.31, *SE* = 0.04, *t*(666) = 7.94, p < .001) (see Supplementary Figure 10). These results suggest that people who attend the mass gathering for the first time may take several days to "warm up" before they feel as transformed as those who are returning.

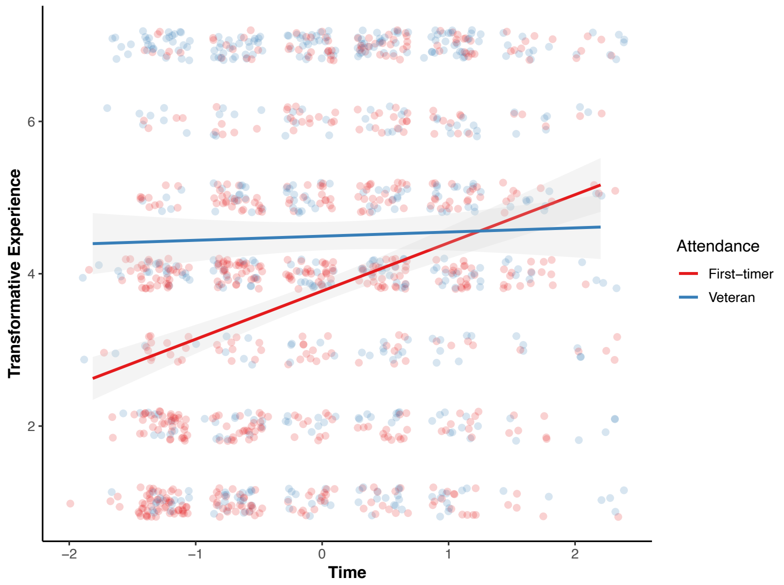

Supplementary Figure 10. Effect of time on reported transformative experience for first-timers versus veterans onsite. Dots represent jittered participant values. Ribbon = *SEM*.

1.10. Other Aspects of Transformative Experience

In addition to the questions regarding transformative experience reported in the main article, we collected several other questions regarding details of people's transformative experiences in order to obtain a more precise picture of their experiences.

1.10.1. Extent. We asked people the extent to which they felt transformed (1 - Minimal or no transformation to 7 - Complete transformation). About 6% of people onsite said they had experienced a “complete transformation.” The extent to which people felt transformed correlated to the overall degree to which they reported both subjective and epistemic transformation, $r(935) = .84$, $p < .001$, $r(924) = .69$, $p < .001$. Extent of transformation increased over time, $B = 0.14$, $SE = 0.03$, $t(912) = 4.50$, $p < .001$, and positively predicted universal connectedness, $B = 0.12$, $SE = 0.03$, $t(861) = 3.59$, $p < .001$.

1.10.2. Valence. We asked people how good and the experience felt (Not at all good to Extremely good) and how bad the experience felt (Not at all bad to Extremely bad). Of the people who had at least “somewhat” of a subjective transformation, the overwhelming majority of them said this was a positive experience, with 34% saying the experience was “extremely good” and 14% saying the experience felt even somewhat bad (about 1% said the experience was “extremely bad”). The longer people were at the event, the better the transformation felt, $B = 0.14$, $SE = 0.03$, $t(880) = 4.67$, $p < .001$. Positive valence was related to universal connectedness, $B = 0.10$, $SE = 0.04$, $t(850) = 2.71$, $p = .007$, but not to moral expansion, $p = .40$.

1.10.3. Moral transformation. We asked participants whether the experience resulted in a perceived change in moral values (“Not at all” to “Absolutely”). Self-reported moral transformation showed significant increases over time, $B = 0.06$, $SE = 0.03$, $t(1119) = 1.96$, $p = .050$. Thus the moral expansion associated with transformative experiences may also be accompanied by a subjective sense of moral change.

1.11. Other Model Specifications

Following past research[6], we conceptualized transformative experiences as epistemic discoveries that precipitate shifts in one’s preferences, values, or beliefs. In other words, such

experiences generate new knowledge that could not have been anticipated before the experience, and this new knowledge is predicted to subsequently impact values and behavior. In the case of transformative experiences at mass gatherings, we hypothesized that transformative experiences precipitate increased feelings of universal connectedness, which in turn predicts increases in moral expansion. Accordingly, we built our mediation models such that transformative experience precedes universal connectedness and moral expansion, rather than the other way around. Thus, in our analyses (see Figure 5), we modeled transformative experience as being directly associated with time onsite and predicting universal connectedness and moral expansion. On the other hand, it is possible that time onsite leads to increased universal connectedness, which subsequently leads to feelings of personal transformation.

To test this question, we constructed and tested an exploratory mediation model in which time onsite predicted universal connectedness, which subsequently predicted transformative experience and moral expansion. All paths showed significantly reduced model fit than the original model specification. The total indirect effect was no longer significant, $B = 0.00$, $SE = 0.00$, $p = 0.712$, $CI_{95}[0.00, 0.00]$, nor was the effect of time on universal connectedness, $B = -0.02$, $SE = 0.03$, $t(1142) = -0.61$, $p = 0.542$, nor was the effect of transformative experience on moral expansion, $B = 0.01$, $SE = 0.02$, $t(866) = 0.29$, $p = 0.771$. Thus reversing the variable order here does not appear to provide a more robust explanation for the relationships between the variables.

1.12. Group identity fusion.

Past research suggests mass gatherings are associated with a fusing of self- and group-based identities. We considered the alternative possibility that this type of group identity fusion, rather than universal connectedness, explains the relationship between transformative experience

and moral expansion. We measured group identity fusion by asking participants to select from five options the set of overlapping circles that they felt best represented their relationship with the group of people who had attended the same event[7]. Group identity fusion and universal connectedness were positively associated with one another but not identical, $r(891) = .35$, $p < .001$.

We tested a model that substituted group identity fusion for universal connectedness in the primary onsite mediation analysis depicted in Figure 5. Results showed that the total indirect effect (that is, of time onsite to moral expansion through transformative experience and group identity fusion) was no longer significant, $B = 0.002$, $SE = 0.002$, $p = 0.155$, CI_{95}[-0.001, 0.006]. Transformative experience positively predicted group identity fusion, $B = 0.15$, $SE = 0.02$, $t(718) = 8.14$, $p < .001$. However, group identity fusion did not significantly predict moral expansion, $B = 0.06$, $SE = 0.04$, $t(809) = 1.50$, $p = .135$. To confirm that universal connectedness, as opposed to group identity fusion, was primarily associated with moral expansion, we ran a regression model that included both of these variables as predictors of moral expansion, which allowed us to determine which played a larger role by allowing them to compete directly for variance. In this model, universal connectedness remained a significant predictor of moral expansion, $B = 0.09$, $SE = 0.03$, $t(798) = 3.09$, $p = .002$, while group identity fusion no longer predicted moral expansion, $B = 0.04$, $SE = 0.04$, $t(798) = 0.85$, $p = .398$, suggesting that moral expansion at these mass gatherings is due more to feelings of universal connectedness than to group identity fusion.

1.13. Predictors of Transformative Experience

Here we examine several variables that might help to explain how mass gathering participation might engender transformative experiences. In the following analyses, we specified models that took a variety of behaviors and tested via mediation whether they helped account for

the increase in transformative experience over time.

1.13.1. New friends. First, we tested whether increased connection to people in the event community was associated with transformative experience over time. Participants indicated how many new friends they had made thus far at the event (0 to More than 50). A mediation model with time as the predictor, new friends as the mediator, and transformative experience as the dependent variable showed a significant indirect effect, $B = 0.06$, $SE = 0.009$, $p < 0.001$, $CI_{95}[0.042, 0.077]$, suggesting that making new social connection partially accounted for the increase in the prevalence of transformative experience over time. However, the direct effect from time onsite to transformative experience remained significant in the model, $B = 0.122$, $SE = 0.009$, $p < 0.001$, $CI_{95}[0.042, 0.077]$, suggesting that this behavior did not entirely explain this relationship.

1.13.2. Giving and receiving gifts. Next we tested whether participation in prosocial activities helped to account for the increase in transformative experience over time. Participants were asked how many gifts they had given (0 to More than 50) and received (0 to More than 50) thus far at the event. We then examined the indirect effect of time on transformative experience through each of these variables respectively. Results showed significant indirect effects of both giving, $B = 0.071$, $SE = 0.012$, $p < 0.001$, $CI_{95}[0.048, 0.094]$, and receiving, $B = 0.068$, $SE = 0.011$, $p < 0.001$, $CI_{95}[0.046, 0.09]$, gifts. However, in both cases ($B = 0.108$, $SE = 0.032$, $p = 0.001$, $CI_{95}[0.045, 0.171]$; $B = 0.112$, $SE = 0.032$, $p = 0.001$, $CI_{95}[0.047, 0.09]$) the direct effects from time to transformative experience remained significant when including these behaviors in the model, suggesting that these behaviors did not entirely account for the relationship between time and transformative experience.

1.13.3. Dancing (behavioral synchrony). Finally, following research suggesting that behavioral synchrony can engender generalized prosocial behavior8, we examined whether self-reported frequency of dancing accounted for the relationship between time and transformative experience. Participants indicated how many separate occasions they had danced at the event name so far (0 to More than 20). As with the other variables, analysis showed a significant indirect effect, $B = 0.041$, $SE = 0.009$, $p < 0.001$, $CI_{95}[0.023, 0.059]$, yet the direct effect from time onsite to transformative experience remained significant when including it in the model, $B = 0.143$, $SE = 0.031$, $p < 0.001$, $CI_{95}[0.023, 0.06]$, suggesting it did not entirely account for the effect.

3. Supplementary Tables

3.1. Event Descriptions

Supplementary Table 4. *Site Descriptions*

**Event Name**	**Description**
Burning Man	Burning Man is a large event held annually in Nevada's Black Rock Desert. The event attracts upwards of 70,000 participants, features a variety of artistic installations and participant-generated events, and is described as "a temporary metropolis dedicated to community, art, self-expression and self-reliance". (https://burningman.org/event/)
Burning Nest	Burning Nest is an officially recognized regional event associated with Burning Man that takes place in the UK every year. The program is entirely participant-generated, and centers around art, music and a variety of workshops. https://www.burningnest.co.uk/
Lightning in a Bottle	Lightning in a Bottle is a transformative music and arts festival held annually in Central California. The event is centered around electronic dance music, ecological sustainability, community building, workshops and yoga. https://en.wikipedia.org/wiki/Lightning_inaBottle
Dirty Bird Campout	Dirty Bird Campout is a music festival held annually in Northern California. The event centers around the principles of judgement-free self-expression, comradery and creativity. https://www.dirtybirdcampout.com/west/what-is-dirtybird-campout
Latitude	Latitude is a music and arts festival held annually in the UK. It includes a comprehensive bill of musicians, bands and artists across four stages. The festival comprises elements of theatre, art, comedy, cabaret, poetry, politics, dance and literature (https://en.wikipedia.org/wiki/Latitude_Festival).

3.2. Demographic Information

Note: Blank cells indicate no data collected.

Supplementary Table 5.
Pre-test

	Burning Man 2015	Burning Man 2016	Burning Nest 2016	Latitude 2016	Dirty Bird Campout 2016	Lightning in a Bottle 2017
**Gender (M/F/Other)**	139/101/3	208/141/8				
**Age (M, (SD))**	40 (13.4)	44 (11.6)				
**Education (% college)**		35.2				
**Income (% > 50k)**		60.1				
**Religious (% > "somewhat")**		18.7				

Supplementary Table 6.
Onsite

	Burning Man 2015	Burning Man 2016	Burning Nest 2016	Latitude 2016	Dirty Bird Campout 2016	Lightning in a Bottle 2017
**Gender (M/F/Other)**	80/51/0	191/139/8	48/47/5	75/91/2	41/41/1	191/193/8
**Age (M, (SD))**	37.1 (13.5)	36.7 (11.8)	32.1 (6.9)	36.1 (14.3)	31.4 (7.0)	25.6 (5.9)
**Education (% college)**	75.4	76.7	42.0	27.9	66.2	48.6
**Income (% > 50k)**	51.1	55.3	14.1	15.1	50.3	27.2
**Religious (% > "somewhat")**	25.7	25.1	18.0	18.4	32.5	28.4

Supplementary Table 7.
Immediate Follow-up

	Burning Man 2015	Burning Man 2016	Burning Nest 2016	Latitude 2016	Dirty Bird Campout 2016	Lightning in a Bottle 2017
**Gender (M/F/Other)**		942/789/28	16/10/2	13/8	5/4	25/15/1
**Age (% over 40)**		49.4	31.5	38.1	33.3	2.5
**Education (% college)**		75.7	57.1	42.8	44.4	60
**Income (% > 50k)**		59.4	10.7	23.8	33.3	12.5
**Religious (% > "somewhat")**		15.4	14.3	23.8	22.2	17.5

Supplementary Table 8.
Six Month Follow-up

	Burning Man 2015	Burning Man 2016	Burning Nest 2016	Latitude 2016	Dirty Bird Campout 2016	Lightning in a Bottle 2017
**Gender (M/F/Other)**	122/100/5	227/200/10	8/7/2	9/4		10/4/0
**Age (M, (SD))**	45.7 (15.3)	44.4 (14.1)	31.3 (3.6)	35 (17)		23.6 (2.5)
**Education (% college)**		82.8	82.4	23		57
**Income (% > 50k)**		62.5	5.9	14		12.5
**Religious (% > "somewhat")**		14.9	5.9	23		50

Supplementary Table 9. *Comparison of total onsite sample characteristics to US, UK, and Mechanical Turk Average*[9]

	**Onsite**	**US**[10]	**UK**[11]	**Mechanical Turk**[12,13]
**Gender**				
M	51.8	49.2	51	55.6
F	46.4	50.8	49	44.4
Other	1.8	NA	NA	NA
**Age**				
18 - 29	52.7	16.4[14]	15.2	34.0
30 – 49 years	35.6	25.6	26.2	38.1
50+ years	11.7	34[15]	39	13.6
**Education**				
High school	6	28	65.6	35.2
Some college/2 year degree	36.5	30.5	21	34.4
College degree	37.9	25.8	21	49.9
Advanced degree	19.1	10.2	4	11.4
**Income**[16]				
Under 35k	51.8	35.7	71	41.5
35-50k	11.7	11.6	15	17.7
50-80k	16.3	14	9	19
80-99k	5.9	6.9	3	10.5
100k+	14	12.6	2	11.4

SUPPLEMENTARY REFERENCES

1. Forstmann, M., Yudkin, D. A., Prosser, A. M., Heller, S. M., & Crockett, M. J. (2020). Transformative experience and universal connectedness mediate the mood-enhancing effects of psychedelic use in naturalistic settings. *Proceedings of the National Academy of Sciences*, *117*(5), 2338-2346.
2. Rachlin, H., & Jones, B. A. (2008). Social discounting and delay discounting. *Journal of Behavioral Decision Making*, *21*(1), 29-43.
3. https://aspredicted.org/blind.php?x=u5bg7c
4. Stef van Buuren, Karin Groothuis-Oudshoorn (2011). mice: Multivariate Imputation by Chained Equations in R. Journal of Statistical Software, 45(3), 1-67.
5. Shister, N. (September 2020). Is Virtual Burning Man the Internet's Ultimate Test? *New York Times.* Retrieved from https://www.nytimes.com/2020/09/03/opinion/virtual-burning-man-2020.html.
6. Paul, L. A. (2014). *Transformative experience*. OUP Oxford.
7. Swann Jr, W. B., Gómez, Á., Seyle, D. C., Morales, J., & Huici, C. (2009). Identity fusion: The interplay of personal and social identities in extreme group behavior. *Journal of Personality and Social Psychology*, *96*(5), 995.
8. Reddish, P., Tong, E. M., Jong, J., Lanman, J. A., & Whitehouse, H. (2016). Collective synchrony increases prosociality towards non-performers and outgroup members. *British Journal of Social Psychology*, *55*(4), 722-738.
9. Data compiled from external sources is approximate. Percentages do not total 100 because they exclude people under 18
10. https://www.census.gov/programs-surveys/acs
11. https://www.statista.com/statistics/275394/median-age-of-the-population-in-the-united-kingdom/
12. https://journals.plos.org/plosone/article?id=10.1371/journal.pone.0198835
13. https://www.ncbi.nlm.nih.gov/pmc/articles/PMC5675003/
14. https://www.marketingcharts.com/featured-30401
15. https://www.jchs.harvard.edu/sites/default/files/jchs-housing_americas_older_adults_2014-ch2_0.pdf
16. Income for UK is in approximate pound equivalent

Let's Do Some Science!

STEP ONE: FIND YOUR TICKETS

You have been given a GOLD ENVELOPE! The envelope contains 10 tickets. Take them out of the envelope now. If the envelope contains fewer or more than 10 tickets, please notify the nearest volunteer.

You can redeem these tickets at your leisure for MYSTERY PRIZES. What exactly are these mystery prizes? Well, if we told you, then it wouldn't be a mystery, would it? Rest assured these prizes are worth your while. We have gone to great lengths to assemble a large assortment of items that festivalgoers find valuable, useful, or weird. However, some of these items are more desirable than others. The least desirable items cost only one ticket, but more desirable items can cost up to 10 tickets.

STEP TWO: CHOOSE HOW MANY TICKETS TO KEEP AND GIVE

Now you have a choice: You can keep all 10 tickets for yourself. Or, you may give as many tickets as you would like to an anonymous stranger by placing them back in the envelope and returning the envelope to us. We will then give these tickets to another person at the festival. This person will not be told anything about where the tickets came from; they will simply get to enjoy the tickets (and the prizes) for themself.

This choice is yours, and yours alone. Your choice is COMPLETELY CONFIDENTIAL. Our volunteers will not know how many tickets you decided to keep or give because the tickets will be concealed in the envelope. The tickets are redeemable at another location that is not visible from here. When you go to redeem your tickets, the volunteer who takes your tickets won't know whether you decided to keep these tickets for yourself, or received them from someone else. And if you do decide to give some tickets to someone else, the person who receives them won't know anything about you or the choice you made. Your choice will be known only to you. If you have any questions, you can ask one of our volunteers anytime

STEP THREE: PUT THE TICKETS IN THE ENVELOPE

Go ahead and make your choice. Decide how many tickets (if any) you want to give to another person and put them back in the envelope. You can anonymously deposit this envelope into a box with one of our volunteers.

Next, take the tickets you're going to keep and put them somewhere safe. We will explain where to redeem them at the end of the study.

STEP FOUR: IF YOU'RE HAPPY AND YOU KNOW IT, CLAP YOUR HANDS!

STEP FIVE: ANSWER THE QUESTIONS ON THE NEXT PAGES

After you've decided what to do with the tickets, please complete the rest of these questions. When you've finished please return your survey to one of our volunteers, and deposit your envelope in the box at the survey station.

And now, some questions for you!

Survey Matcher

This section is so that we can match you with any other data you have given us while still making sure your responses remain completely ***anonymous*** *and* ***confidential****.*

Please write the first three letters of the first road on which you ever lived (e.g., "MAR"): ___________

Please enter the 2 digit calendar day of your birthday (e.g., "08"): ________________

Please enter the last two letters of your mother's maiden name (e.g., "ES"): ______________________

****

Have you heard anything about this study before today? Circle one: Y N

If yes, what have you heard? ____

Please circle the image that best represents your current mood.

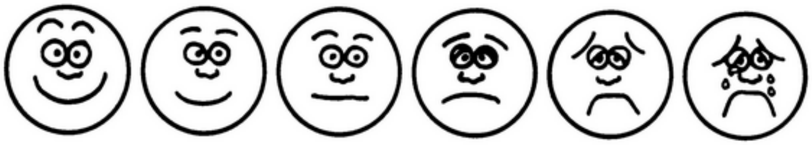

What year were you born? ______________________

What is your gender? M F Both/Neither/Fluid

Where do you usually reside when not in Black Rock City? ____

(*Please indicate your Country and, if US, your state and zip code)*

Did you vote in any of the last four US elections? Circle all that apply. *If you are not eligible to vote in the US, circle "Not Eligible". If you are eligible but did not vote in any of the last 4 elections, circle "Did Not Vote".*

2014 2012 2010 2008 Not Eligible Did Not Vote

What was the first language you learned and still use (i.e. native language)? Circle one:

English Other: ___________________________________

With which political party are you currently affiliated in the United States, if any? (check one)

- ☐ Democratic Party
- ☐ Republican Party
- ☐ Libertarian Party
- ☐ Green Party
- ☐ American Independent Party (formerly America's Party)
- ☐ Other US Party: __________________________
- ☐ None, I choose to remain unaffiliated

What is the highest level of education you have completed?

- ☐ High school
- ☐ Some college
- ☐ 2 year degree
- ☐ 4 year degree
- ☐ Postgraduate or professional degree

Politically, how liberal (left-wing) or conservative (right-wing) are you?

0	0	0	0	0	0	0
Extremely Liberal			Moderate, Middle of Road			Extremely Conservative

Please indicate the category that describes the total amount of income you earned in 2014:

- ☐ Under $5k
- ☐ $5k - $10k
- ☐ $11k - $15k
- ☐ $16k - $25k
- ☐ $26k - $35k
- ☐ $36k - $50k
- ☐ $51k - $65k
- ☐ $66k - $80k
- ☐ $81k - $100k
- ☐ Over $100k

Please rate how religious you consider yourself to be.

0	0	0	0	0	0	0
Not religious			Somewhat			Very religious

How many times have you previously attended Burning Man, excluding this year?

- ☐ 0
- ☐ 1
- ☐ 2
- ☐ 3-4
- ☐ 5-7
- ☐ 8+

When did you **arrive** at Burning Man?

- ☐ Before Friday 26th August
- ☐ Saturday 27th August
- ☐ Sunday 28th August
- ☐ Monday 29th August
- ☐ Tuesday 30th August
- ☐ Wednesday 31st August
- ☐ Thursday 1st September
- ☐ Friday 2nd September

When do you plan to **depart** Burning Man?

- ☐ Before Thursday 1st September
- ☐ Thursday 1st September
- ☐ Friday 2nd September
- ☐ Saturday 3rd September
- ☐ Sunday 4th September
- ☐ Monday 5th September
- ☐ After Monday 5th September

For how many days have you been at Burning Man? _______________

How many people at Burning Man did you already know upon arriving?

- ☐ None
- ☐ 1-2
- ☐ 3-5
- ☐ 6-10
- ☐ 11-20
- ☐ 21-50
- ☐ More than 50

How many new friends have you made at Burning Man since you arrived?

- ☐ None
- ☐ 1-2
- ☐ 3-5
- ☐ 6-10
- ☐ 11-20
- ☐ 21-50
- ☐ More than 50

Approximately how many times have you *given* someone a gift at Burning Man so far this year?

- ☐ None
- ☐ 1-2
- ☐ 3-5
- ☐ 6-10
- ☐ 11-20
- ☐ 21-50
- ☐ More than 50

Approximately how many times have you *received* a gift at Burning Man so far this year?

- ☐ None
- ☐ 1-2
- ☐ 3-5
- ☐ 6-10
- ☐ 11-20
- ☐ 21-50
- ☐ More than 50

Approximately how many times have you handled money at Burning Man so far this year?

- ☐ None
- ☐ 1-2
- ☐ 3-5
- ☐ 6-10
- ☐ 11-15
- ☐ 16-20
- ☐ More than 20

On how many separate occasions have you danced at Burning Man so far this year?

- ☐ None
- ☐ 1-2
- ☐ 3-5
- ☐ 6-10
- ☐ 11-15
- ☐ 16-20
- ☐ More than 20

Have you attended any other Burner events in the past (excluding Burning Man)? Circle one: Y N

The following questions are about your personal experiences at Burning Man.

Have you had a transformative experience while at Burning Man?

0	0	0	0	0	0	0
Not at all			Somewhat			Absolutely

What was the extent of this transformation?

0	0	0	0	0	0	0
Minimal or no transformation						Complete transformation

How good did this transformation feel?

0	0	0	0	0	0	0
Not at all good						Extremely good

How bad did this transformation feel?

0	0	0	0	0	0	0
Not at all bad						Extremely bad

Did you participate expecting this transformative experience?

0	0	0	0	0	0	0
Not at all			Somewhat			Absolutely

Did you participate desiring this transformative experience?

0	0	0	0	0	0	0
Not at all			Somewhat			Absolutely

So far, has your experience caused you to significantly change your moral values?

0	0	0	0	0	0	0
Not at all			Somewhat			Absolutely

Please circle the image that best describes your current relationship with other human beings, in general.

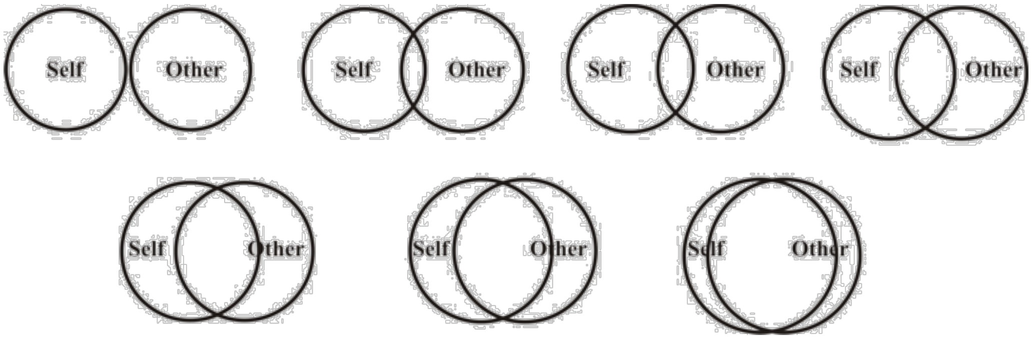

To what extent have you experienced the following changes at Burning Man this year?

	Not at all	0	0	Somewhat	0	0	Completely
I perceived something new about myself	☐	☐	☐	☐	☐	☐	☐
I perceived something new about others	☐	☐	☐	☐	☐	☐	☐
I perceived something new about reality	☐	☐	☐	☐	☐	☐	☐
I felt more socially connected to others	☐	☐	☐	☐	☐	☐	☐
I felt more spiritually connected to something larger than myself (a higher power, nature, God)	☐	☐	☐	☐	☐	☐	☐
I felt more socially connected to something larger than myself (community, culture, history)	☐	☐	☐	☐	☐	☐	☐
I felt as though my "self" was dissolved	☐	☐	☐	☐	☐	☐	☐
I felt like layers have been peeled back to reveal my true self	☐	☐	☐	☐	☐	☐	☐
I felt like I could better express my true self	☐	☐	☐	☐	☐	☐	☐
I feel like I am a different person than I was before	☐	☐	☐	☐	☐	☐	☐

We are curious about a special kind of transformative experience: an experience that changes you so profoundly that you come out of the experience radically different than you were before the experience. This transformation

may have been so profound that you could not have known what the experience or change would be like before going through it.

Did you have *this* type of transformative experience while at Burning Man?

0	0	0	0	0	0	0
Not at all			Somewhat			Absolutely

If you have had this type of transformative experience, are you glad that you did? In other words, in retrospect, was the transformation desirable?

0	0	0	0	0	0	0
Not at all			Somewhat			Absolutely

Please circle the image below that you feel best represents your relationship with <u>other Burners</u> as a group.

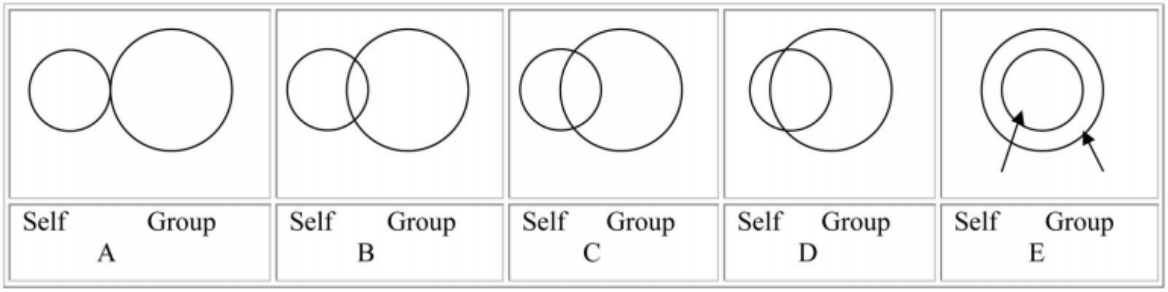

To what extent do you agree with the following statements?

I assume that people have only the best intentions.

0	0	0	0	0	0	0
Not at all			Somewhat			Absolutely

When someone does me a favor I am willing to return it.

0	0	0	0	0	0	0
Not at all			Somewhat			Absolutely

Please think about what you would do in the following situation.
Suppose you were given $100, and had the opportunity to give any amount of money from $0 to $100 to a random stranger. This person would never know who gave them the money, and would not know how much money you were originally given.

If you were given $100, how much (from 0-$100) would you give to a random stranger? _________

THESE QUESTIONS ARE ABOUT DRUGS!

Please note that the following section is entirely optional. The answers you provide here, as with the entire survey, will completely anonymous. Any results will only be reported in aggregate, without any identifying information from participants.

Are you currently under the influence of any substance? Circle one: Y N

Have you used any substances in the last 24 hours? Circle one: Y N

Have you used any substances at all at Burning Man this year? Circle one: Y N

Have you used any substances, for the first time in your life, at Burning Man this year? Circle one: Y N

In the table below, for each category of substances, please indicate whether you are currently under the influence of that substance; whether you have taken that substance within the last 24 hours or the last week; and whether you have taken that substance for the first time this week.

	Currently under the influence	Taken in last 24 hours	Taken at any time this week	Taken for the first time this week
Alcohol	❑	❑	❑	❑
Nicotine	❑	❑	❑	❑
Cannabis products (e.g., weed, THC, CBD, hemp oil)	❑	❑	❑	❑
Hallucinogens (e.g., psilocybin, LSD, salvia, mescaline, DMT)	❑	❑	❑	❑
Euphorics (e.g., MDMA, Molly, Kratom)	❑	❑	❑	❑
Stimulants (e.g., cocaine, methamphetamine, ephedrine)	❑	❑	❑	❑
Narcotic Analgesics (e.g., morphine, heroin, oxycodone)	❑	❑	❑	❑
Benzodiazepines (e.g., Valium, Alprazolam [Xanax])	❑	❑	❑	❑
Inhalants (poppers, whip-its, nitrous oxide, glue)	❑	❑	❑	❑
Other	❑	❑	❑	❑

Giving Time to Others

Some people are more socially close to you than others. Imagine that 100 people were lined up in a row according to how socially close they are to you. The diagram below visualizes how this line might look.

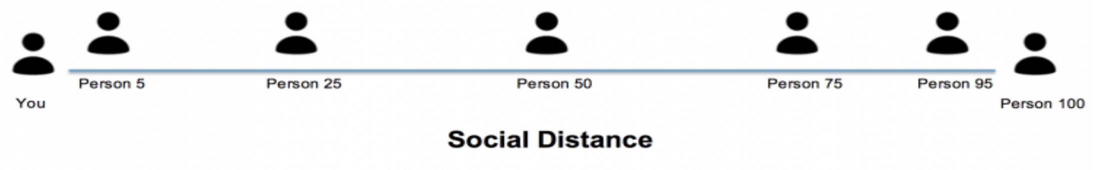

Person 1 is the person you are closest with socially. Person 100 is very socially distant, perhaps a stranger you passed on the street one day. Person 50 could be a distant acquaintance or friend of a friend, someone whose name you may not know.

In the spaces below please enter the initials of representatives for the following social distances. This information is entirely anonymous.

Person 1 ______________________________ Person 5 ______________________________

Person 2 ______________________________ Person 10 ______________________________

Person 3 ______________________________ Person 20 ______________________________

Suppose you were given 14 hours of free time, and had the opportunity to spend any amount of this time (from 0-14 hours) doing a personal favor for each of the persons listed below. Enter your responses in hours, with a maximum of two decimal places. Please treat each person as a separate case.

How many hours would you spend doing a favor for the 1st socially closest person to you? _____ Hours

How many hours would you spend doing a favor for the 2nd socially closest person to you? _____ Hours

How many hours would you spend doing a favor for the 3rd socially closest person to you? _____ Hours

How many hours would you spend doing a favor for the 5th socially closest person to you? _____ Hours

How many hours would you spend doing a favor for the 10th socially closest person to you? _____ Hours

How many hours would you spend doing a favor for the 20th socially closest person to you? _____ Hours

How many hours would you spend doing a favor for the 50th socially closest person to you? _____ Hours

How many hours would you spend doing a favor for the 100th socially closest person to you? _____ Hours

---

THANK YOU FOR PARTICIPATING! PLEASE NOW FIND THE NEAREST VOLUNTEER.
