# Supplementary material for: Prosocial correlates of transformative experiences at secular multi-day mass gatherings
Source: Nat Commun. 2022 May 27;13:2600. doi: 10.1038/s41467-022-29600-1 (PMC9142525; doi:10.1038/s41467-022-29600-1)
Supplement: Supplementary file 3 — Reporting Summary [file 41467_2022_29600_MOESM3_ESM.pdf]

nature research

Corresponding author(s): Daniel Yudkin
Molly Crockett

Last updated by author(s): Jan 24, 2022

Reporting Summary

Nature Research wishes to improve the reproducibility of the work that we publish. This form provides structure for consistency and transparency in reporting. For further information on Nature Research policies, see our Editorial Policies and the Editorial Policy Checklist.

Statistics

For all statistical analyses, confirm that the following items are present in the figure legend, table legend, main text, or Methods section.

n/a	Confirmed	
☐	☒	The exact sample size (n) for each experimental group/condition, given as a discrete number and unit of measurement
☐	☒	A statement on whether measurements were taken from distinct samples or whether the same sample was measured repeatedly
☐	☒	The statistical test(s) used AND whether they are one- or two-sided<br>*Only common tests should be described solely by name; describe more complex techniques in the Methods section.*
☐	☒	A description of all covariates tested
☐	☒	A description of any assumptions or corrections, such as tests of normality and adjustment for multiple comparisons
☐	☒	A full description of the statistical parameters including central tendency (e.g. means) or other basic estimates (e.g. regression coefficient) AND variation (e.g. standard deviation) or associated estimates of uncertainty (e.g. confidence intervals)
☐	☒	For null hypothesis testing, the test statistic (e.g. F, t, r) with confidence intervals, effect sizes, degrees of freedom and P value noted<br>*Give P values as exact values whenever suitable.*
☐	☒	For Bayesian analysis, information on the choice of priors and Markov chain Monte Carlo settings
☐	☒	For hierarchical and complex designs, identification of the appropriate level for tests and full reporting of outcomes
☐	☒	Estimates of effect sizes (e.g. Cohen's d, Pearson's r), indicating how they were calculated

Our web collection on statistics for biologists contains articles on many of the points above.

Software and code

Policy information about availability of computer code

Data collection	Qualtrics 2015 - 2020
Data analysis	R 4.0 (open source); All data were cleaned and analyzed using R data analysis software with packages broom 0.7.11, cowplot 1.1.1, tidyverse 1.3.1, forcats 0.5.1, lavaan, 0.6-9, lm.beta 1.5-1, lme4 1.1-27.1, psych 2.1.9, and sjPlot 2.8.10. Analyses examining behavioral and attitudinal effects across events were performed with a mixed model regression using package lmerTest 3.1-3 in R with event set as random intercept and all other predictors as fixed factors. Mediation analyses were conducted using the lavaan package in R (version .5-23.1097).<br><br>All code is available at https://osf.io/x5uz9/.

For manuscripts utilizing custom algorithms or software that are central to the research but not yet described in published literature, software must be made available to editors and reviewers. We strongly encourage code deposition in a community repository (e.g. GitHub). See the Nature Research guidelines for submitting code & software for further information.

Data

Policy information about availability of data

All manuscripts must include a data availability statement. This statement should provide the following information, where applicable:

- Accession codes, unique identifiers, or web links for publicly available datasets
- A list of figures that have associated raw data
- A description of any restrictions on data availability

Full scripts and (anonymized) data are available at https://osf.io/x5uz9/.

Field-specific reporting

Please select the one below that is the best fit for your research. If you are not sure, read the appropriate sections before making your selection.

☐ Life sciences ☒ Behavioural & social sciences ☐ Ecological, evolutionary & environmental sciences

For a reference copy of the document with all sections, see nature.com/documents/nr-reporting-summary-flat.pdf

Behavioural & social sciences study design

All studies must disclose on these points even when the disclosure is negative.

Study description	A multi-pronged investigation of prosocial change before, during, and after attending one of several multi-day mass gatherings.
Research sample	Overall, the onsite sample included 625 men, 558 women, 32 fluid/other, with a mean age of 32.4 (SD = 11.4) and age range of 17 to 75. Fifty-seven percent had college degrees, and 37% made over $50,000. The sample skewed liberal, with a mean of 2.6 (“somewhat liberal”) on a 7-point scale (1 = “extremely liberal”, 7 = “extremely conservative”). Some differences between the events should be noted. While most events had an average age in the mid-thirties, Lightning in a Bottle attendees were significantly younger, with a mean age of 26. This is also reflected in the fact that fewer of them (47%) had graduated college. The event with the lowest income was Burning Nest (16% making $50,000 or more), as contrasted with Burning Man, with an average of 56% at that income level. Overall, participants in the overall sample were not particularly religious, with a mean of 2.3 out of a religiosity scale of 7. The pre-attendance sample (n = 600) had a makeup of 242 men, 347 women, 11 = other/fluid, Mage = 42.2, SD = 13.5. The immediate followup sample (n = 1,866) had a makeup of 962 men, 818 women, 86 = other/fluid, Mage = 40.1 SD = 12.3. The six month followup sample (n = 697) had a makeup of 311 men, 367 women, 19 = other/fluid, Mage = 44.1, SD = 14.7.
Sampling strategy	We did not have a predetermined sample size as our aim was to collect as many participants as possible at each event location for maximal statistical precision. The sample sizes were sufficient to determine small to medium changes in prosocial orientation over time at multi-day mass gatherings.
Data collection	Field site selection . We identified a set of field sites that varied independently on several features of scientific interest (see Table 1). After identifying two gift-economy field sites (Burning Man and Burning Nest), we then sought other locations that matched these events on critical features. Ultimately, we selected two market economy field sites on the West coast of the United States (Dirty Bird and Lightning in a Bottle) to match the geographical location of Burning Man; we also selected another market-economy event in the UK (Latitude) to match the location of Burning Nest. After we had identified the relevant locations, we reached out to local event organizers to obtain permission to collect data at the events.<br><br>Onsite data collection. At each of the field sites, 6-8 volunteer research assistants with a background in psychology and ethics training in human subjects research aided in data collection. Data collection was performed in consultation with third author S. Megan Heller, a cultural anthropologist with experience in field research at mass gatherings. Research assistants were instructed to recruit participants from a booth set up in well-trafficked event areas. Wearing white lab coats, they approached passersby and asked whether they were interested in taking part in an activity called “Play Games for Science.” Notably, we did not mention transformative experience or prosocial change in our recruitment efforts in an attempt to minimize selection bias. Prior to data collection, all research assistants attended a training where they were instructed in data collection protocol and practiced the protocol on one another. After providing informed consent, participants answered the survey questions either on paper (Burning Man, Lightning in a Bottle, Latitude) or on electronic tablets (Burning Nest, Dirty Bird). Overall, the study took approximately 15 minutes for each participant to complete. Following completion, participants were given an opportunity to collect a prize and thanked for their participation.
Timing	Data collection started on August 1st, 2015 and ended on September 1st, 2020.
Data exclusions	Participants were excluded based on the pre-established procedure of who responded anything other than “0” to the attention check “How many fatal heart attacks have you had?” (n = 26). In addition, we performed a post-hoc exclusion of participants who reported having been at the event for more than 7 days (n = 37, predominantly at Burning Man) since these generally consisted of event organizers, staff and committed event volunteers.
Non-participation	None
Randomization	Any participant who wished to participate was admitted; thus there was no random assignment to condition. Events were treated as random effects. In addition, we controlled for demographics (i.e., gender, age, education, religiosity, and income), and “incidental variables” (i.e., mood, expectations and desires of having a transformative experience, and the use of psychoactive substances [binary-coded as -.5 or .5 and including euphorics, hallucinogens, stimulants, alcohol, narcotics, and cannabis]) in order to additionally account for between-event differences.

Reporting for specific materials, systems and methods

We require information from authors about some types of materials, experimental systems and methods used in many studies. Here, indicate whether each material, system or method listed is relevant to your study. If you are not sure if a list item applies to your research, read the appropriate section before selecting a response.

Materials & experimental systems

n/a	Involved in the study
☒	☐ Antibodies
☒	☐ Eukaryotic cell lines
☒	☐ Palaeontology and archaeology
☒	☐ Animals and other organisms
☐	☒ Human research participants
☒	☐ Clinical data
☒	☐ Dual use research of concern

Methods

n/a	Involved in the study
☒	☐ ChIP-seq
☒	☐ Flow cytometry
☒	☐ MRI-based neuroimaging

Human research participants

Policy information about studies involving human research participants

Population characteristics	Overall, the sample included 645 men, 573 women, 3 fluid/other, with a mean age of 32.5 (SD = 11.4) and age range of 17 to 75. Fifty-seven percent had college degrees, and 37% made over $50,000. The sample skewed liberal, with a mean of 2.6 (“somewhat liberal”) on a 7-point scale (1 = “extremely liberal”, 7 = “extremely conservative”). Some differences between the events should be noted. While most events had an average age in the mid-thirties, Lightning in a Bottle attendees were significantly younger, with a mean age of 26. This is also reflected in the fact that fewer of them (47%) had graduated college. The event with the lowest income was Burning Nest (16% making $50,000 or more), as contrasted with Burning Man, with an average of 56% at that income level. Overall, participants in the overall sample were not particularly religious, with a mean of 2.3 out of a religiosity scale of 7.
Recruitment	At each of the field sites, 6-8 volunteer research assistants aided in data collection. Research assistants recruited participants from a booth set up in well-trafficked event areas with a sign labeled “Play Games for Science.” After providing informed consent, participants answered the survey questions either on paper (Burning Man, Lightning in a Bottle, Latitude) or on electronic tablets (Burning Nest, Dirty Bird). Overall, the study took approximately 15 minutes for each participant to complete. Following completion, participants were given an opportunity to collect a prize and thanked for their participation.<br><br>Because participants volunteered to participate in the study, it is possible that there were selection effects wherein participants who experienced certain psychological effects (e.g., had a transformative experience) were more likely to participate. We discuss this and other potential recruitment biases in the study discussion.
Ethics oversight	The project was approved by the University of Oxford Research Ethics Committee (#MS-IDREC-C1-2015-134).

Note that full information on the approval of the study protocol must also be provided in the manuscript.
